# Effects of superhydrophobic sand mulching on evapotranspiration and phenotypic responses in tomato (*Solanum lycopersicum*) plants under normal and reduced irrigation

**DOI:** 10.1002/pei3.10074

**Published:** 2022-04-07

**Authors:** Kennedy Odokonyero, Adair Gallo, Vinicius Dos Santos, Himanshu Mishra

**Affiliations:** ^1^ Environmental Science and Engineering Program, Biological and Environmental Sciences and Engineering Division King Abdullah University of Science and Technology (KAUST) Thuwal Saudi Arabia; ^2^ Water Desalination and Reuse Center (WDRC) King Abdullah University of Science and Technology (KAUST) Thuwal Saudi Arabia; ^3^ Chemical Engineering Program, Physical Science and Engineering Division King Abdullah University of Science and Technology (KAUST) Thuwal Saudi Arabia

**Keywords:** chlorophyll content index, evapotranspiration, food–water security, mulching, *Solanum lycopersicum*, stomatal conductance, superhydrophobic sand, transpiration efficiency

## Abstract

Irrigated agriculture in arid and semi‐arid regions is a vital contributor to the global food supply. However, these regions endure massive evaporative losses that are compensated by exploiting limited freshwater resources. To increase water‐use efficiency in these giga‐scale operations, plastic mulches are utilized; however, their non‐biodegradability and eventual land‐filling renders them unsustainable. In response, we have developed superhydrophobic sand (SHS) mulching technology that is comprised of sand grains or sandy soils with a nanoscale coating of paraffin wax. Here, we investigate the effects of 1 cm‐thick SHS mulching on the evapotranspiration and phenotypic responses of tomato (*Solanum lycopersicum*) plants as a model system under normal and reduced irrigation inside controlled growth chambers. Experimental results reveal that under either irrigation scenario, SHS mulching suppresses evaporation and enhances transpiration by 78% and 17%, respectively relative to the unmulched soil. Comprehensive phenotyping revealed that SHS mulching enhanced root xylem vessel diameter, stomatal aperture, stomatal conductance, and chlorophyll content index by 21%, 25%, 28%, and 23%, respectively, in comparison with the unmulched soil. Consequently, total fruit yields, total dry mass, and harvest index increased in SHS‐mulched plants by 33%, 20%, and 16%, respectively compared with the unmulched soil. We also provide mechanistic insights into the effects of SHS mulching on plant physiological processes. These results underscore the potential of SHS for realizing food–water security and greening initiatives in arid regions.

## Graphical abstract

We investigated the effects of superhydrophobic sand (SHS) mulch on evapotranspiration in tomato plants (*Solanum lycopersicum*) grown in percival growth chambers forover 98 days ‐ from saplings to fruition ‐ under normal and reduced irrigation regimes (i.e., 100% and 50% of field capacity, respectively). A broad set of experimental techniques were utilizedto pinpointwhy SHS dramatically improved plant health and fruit yield.

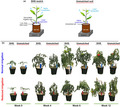

## 1 INTRODUCTION

Irrigated agriculture is a crucial contributor to global food security. Of the total cultivated land only ~20% is irrigated, and yet it contributes a disproportionately high (30%–40%) amount to the global food production (World Water Assessment Programme, 2009). Globally, many arid and semi‐arid regions have emerged as agricultural hubs by exploiting freshwater resources. For instance, in the last 50 years, irrigated agriculture footprints in India, China, and Brazil have expanded by 30% (Jain et al., 2020), 52% (Zhu et al., 2013), and 52% (Carvalho et al., 2020), respectively. Most arid regions are characterized by dry winds and high temperatures that lead to substantial evaporative and transpiration losses (Al‐Naizy & Simonet, 2012; Balugani et al., 2017). These losses are compensated via irrigation and due to the giga‐scale of agricultural operations, irrigation claims ~70% of annual freshwater consumption worldwide (Boretti & Rosa, 2019; Globalagriculture.Org; Koech & Langat, 2018; OECD.Org). Due to the slow recharge of groundwater in arid regions and additional constraints posed by environmental pollution, the climate change, and the increasing use of water in industrial and domestic contexts, ensuring food–water–climate security has emerged as one of the most formidable global challenges for the 21st century (Famiglietti, 2014; Scanlon et al., 2012; Steward et al., 2013; Watto, Bashir, & Niazi, 2018; Watto, Mugera, et al., 2018). Therefore,sustainable technologies for enhancing water‐use efficiency in arid land agriculture are needed urgently.

When water is applied to the topsoil, it is lost via evaporation, transpiration, and percolation (Hillel, 2012; Sutanto et al., 2012). Unlike evaporation and percolation, transpiration is crucial in the photosynthetic process and maintaining the optimal temperature required for plants' metabolic processes (Grill & Ziegler, 1998; Hetherington & Woodward, 2003). Transpiration thus cannot be reduced under the constraint of high crop productivity (without genetic engineering). Percolation could be reduced by applying a water barrier layer beneath the plant root zone, but this process is labor‐intensive and expensive (Nkurunziza et al., 2019). Thus, curtailing evaporative loss of water from the soil is the most prudent approach for enhancing water‐use efficiency in arid lands.

Evaporation and transpiration constitute evapotranspiration (ET) (Hatfield & Dold, 2019; Rawitz & Hadas, 1994). The importance of transpiration is reflected in the water‐use efficiency of plants, that is, the ratio of the total biomass (root and shoot) formed to the cumulative water transpired (Kadam et al., 2015), and the transpiration efficiency (TE), that is, the ratio of the shoot biomass formed to the cumulative water transpired (Vadez et al., 2014). The TE represents an aspect of water‐use efficiency that depends on the water‐conducting (i.e., hydraulic) potential of the plant to facilitate shoot physiological processes that enhance biomass under different soil water scenarios (Vadez et al., 2014). Therefore, TE is of interest in water‐scarce environments where crops are most sensitive to soil moisture (Condon et al., 2002; Sinclair, 2012). Transpiration also influences plants' reproductive efficiency, defined by the harvest index (HI), that is, the ratio of total yield to total vegetative biomass produced, pinpointing the plant resources invested in yield production (Porker et al., 2020; Unkovich et al., 2010).

As noted above, evaporation is the wasteful component of ET, which should be minimized, especially in arid regions. In this context, mulching refers to the application of a vapor diffusion barrier on the topsoil to curtail water evaporation, and it has been proven to enhance the soil moisture content and provide enhanced transpiration (Farzi et al., 2017; Moitra & Sarkar, 1996; Zhang et al., 2018), plant biomass, and yields (Mukherjee et al., 2010; Ramalan & Nwokeocha, 2000; Zhang, Xiong, et al., 2017). Mulching has also been demonstrated to enhance leaf chlorophyll content (Wang et al., 2015), photosynthesis (Niu et al., 2020; Zhang et al., 2019), and TE (Balwinder‐Singh et al., 2011). It also improves plant root growth and root architectural and anatomical properties such as root diameter and late xylem vessel diameter, which improve water and nutrient uptake from the soil (Larsson & Jensen, 1996; Zhan et al., 2019). Low‐density polyethylene sheets of ~0.1 mm thickness have been used extensively for mulching in developed countries (Kasirajan & Ngouajio, 2012). Their application in the developing world is also on the rise, for example, consumption in China is set to exceed 2 million tons per year by 2024 (Liu et al., 2014; Wang et al., 2013). Despite their benefits (Balwinder‐Singh et al., 2011; Li et al., 2004; Yin et al., 2019; Zhang et al., 2019; Zhang, Wei, et al., 2017), plastics are non‐biodegradable, and their eventual disposal into landfills is unsustainable (Halley et al., 2001; Kasirajan & Ngouajio, 2012). Recent findings on the leaching of phthalates from plastic mulches into soils and their adverse effects on the soil quality and microbial activity over the long term are also concerning (Shah & Wu, 2020). Therefore, sustainable mulching technologies are needed.

We have recently developed a nature‐inspired mulching technology–– superhydrophobic sand (SHS) (Gallo et al., 2022; Mishra et al., 2017)––that is comprised of common sand grains or sandy soils coated with a nanoscale layer of paraffin wax. The micro‐ and nanoscale surface roughness of the sand grains (size distribution: 100–700 μm) and the hydrophobic nature of wax give rise to superhydrophobicity (Arunachalam et al., 2019; Arunachalam et al., 2020; Mahadik et al., 2020; Odokonyero et al., 2021). When laid on the topsoil with a sub‐surface irrigation system, a 5–10 mm‐thick SHS layer prevents capillary rise of water (Das et al., 2019; Domingues et al., 2018) and insulates the wet soil from solar radiation and dry air, thereby acting as a diffusion barrier for the water vapor (Gallo Jr et al., 2021). As water evaporation is arrested, more water is available in the plant root region, which boosts plant health in arid regions. In fact, our multi‐year field trials of SHS on tomatoes (*Solanum lycopersicum*), wheat (*Triticum aestivum*), and barley (*Hordeum vulgare*) under arid land conditions in western Saudi Arabia have revealed significant enhancements in plant growth and yields (Gallo et al., 2022). Compared with plastic mulches, SHS is environmentally benign as it is made from sand/sandy soil and paraffin wax, and the latter is degraded by soil microbes (Marino, 1998; Roper, 2004). After about 9 months of SHS application, paraffin wax is degraded due to microbial activity and solar radiation and SHS gets incorporated in the soil, obviating land‐filling; additionally, there are no detectable effects on soil microbial compositions (Gallo et al., 2022). Despite these promising field results, quantitative insights into the effects of SHS on ET under varying irrigation scenarios as well as phenotypic traits responsible for yield enhancement are lacking.

In response, here, we quantify the effects of SHS mulching on tomato plants in growth chambers and pinpoint ET dynamics and phenotypic responses in terms of the following traits: plant height, stomatal conductance, stomatal pore size (aperture), leaf chlorophyll content, fruit yield, HI, TE, fresh mass, dry mass, xylem vessel, and root diameter. We investigate these effects of SHS mulching under normal (**N**) and reduced (**R**) irrigation scenarios, that is, 100% and 50% of pot capacity, respectively.

## 2 MATERIALS AND METHODS

### 2.1 Plants and SHS mulch

Tomato plants (*S. lycopersicum*), Seminis variety (St. Louis, Missouri, US), were purchased from local seed stores in Jeddah, Saudi Arabia. SHS was manufactured using common silica sand and paraffin wax following the optimized protocol detailed in our previous work (Gallo et al., 2022; Mishra et al., 2017). Briefly, sand grains with a size distribution ranging from 100 to 700 μm were utilized. First, paraffin wax was dissolved in an organic solvent (hexane) in a rotary evaporator and the sand was added. The mass ratio of wax: sand was 1:600. Our process led to a conformal coating of wax on each grain. The solvent was then recovered by decreasing the pressure and increasing the temperature and condensed for reuse, leaving behind SHS; resulting SHS grains had a 20 nm‐thick wax coating.

### 2.2 Plant growth conditions, treatments, and experimental design

Tomato seeds were sown in plastic trays using a potting mix from Stender AG and grown in growth chambers (Percival^®^ Scientific chamber, PGC‐6L, Geneva Scientific LLC). After 4 weeks, the seedlings were transplanted into pots 1870 cm^3^ in volume (15 cm top diameter × 10.5 cm bottom diameter × 14.5 cm height) containing approximately 2.4 kg of local sandy soil. The pots were watered via sub‐surface irrigation to the following levels every 2 days: 100% pot capacity for **N** irrigation (i.e., the maximum soil moisture content after drainage of excess water from fully saturated potted soil) and 50% of this pot capacity for **R** irrigation. The irrigation level of each pot was determined gravimetrically. Thirty‐two pots were prepared, including 16 with plants and 16 without plants, the latter of which were used to quantify evaporative losses.

The pots were separated into two groups: those mulched with the SHS and the unmulched ones. In each group, half of the pots were subjected to **N** irrigation while the other half was subjected to **R** irrigation. The soil mulching entailed a 1.0‐cm thick layer of SHS on the potting soil (approximately 265 g SHS/pot). Thus, there were four treatment combinations (SHS‐**N**, unmulched soil‐**N**, SHS‐**R**, and unmulched soil‐**R)**, each with four replicates with and without plants. All pots were subjected to complete randomization in a 2 × 2 factorial design involving two experimental factors (i.e., soil mulching and irrigation regime), each with two levels . The growth chamber consisted of two levels (tier‐1 and tier‐2) with identical environmental conditions and pots were completely randomized in both tiers. During the growth period, nutrient solutions were applied to each pot every 2 weeks at the following rates per kg of dry soil: 200 mg N/kg, 250 mg P/kg, 200 mg K/kg, 150 mg Ca/kg, 30 mg S/kg, 2 mg Cu/kg, 4 mg Zn/kg, 3 mg Mn/kg, 0.5 mg B/kg, and 0.25 mg Mo/kg. The plants were grown for 98 days under a 14/10 h light/dark photoperiod using fluorescent lighting at 350 μmol‐m^−2^‐s^−1^ of photosynthetically active radiation, a day/night temperature of 28/20 ± 2°C, and 40/60 ± 2% day/night relative humidity; air circulation inside the chamber consists of a gentle horizontal air flow. During the study period, meteorological conditions at the KAUST field (22°18′12.4″N 39°06′41.3″E) consisted of 27.7/18.4 ± 6°C average maximum and minimum air temperatures; 35%–74% relative humidity, 0.27 mm/day average rainfall, 252 Ly solar radiation, and average wind speed of 5.8 kmph (Source: KAUST Weather Station).

### 2.3 Evapotranspiration

ET was partitioned into evaporation and transpiration by performing gravimetric measurements of pots every 2 days until the final harvest. The water loss from each pot was monitored, and water was added to compensate for the loss. The daily ET was recorded as the total water lost from each pot containing plants between the time of irrigation to the time of weighing as:

(1) $$\begin{array}{cc} \mathrm{ET}=\; & \left(\text{Initial weight of}\;\mathrm{pot}\;\text{with plant}-\text{final weight of}\;\mathrm{pot}\;\text{with plant}\right)/\\ & \left(\text{time between measurements}\right). \end{array}$$

### 2.4 Evaporation

Here, we consider that the evaporative loss of water from the pots with plants is the same as that in the pots without plants. The daily water loss through evaporation was then estimated from pots without plants as:

(2) $$\begin{array}{c} \text{Evaporation}=\left(\text{Initial weight of}\;\mathrm{pot}\;\text{without plant}-\text{final weight of}\;\mathrm{pot}\;\text{without plant}\right)/\\ \left(\text{time between measurements}\right). \end{array}$$

### 2.5 Transpiration

The daily transpiration was determined as:

(3) $$\text{Transpiration}=\left(\mathrm{ET}-\text{evaporation}\right).$$

### 2.6 Soil water content

Soil water content (SWC; i.e., the pot capacity) was determined gravimetrically. Potting soils each of known mass (i.e., mass of pot plus soil) were fully saturated with water and allowed to drain freely overnight. After 16 h, the final weight of the pot assembly was determined, ensuring that no excess water was still dripping from the pot. The difference between the initial and final pot weight represented the total SWC of the pot (i.e., 100% pot capacity). Thus, we maintained the SWC at a relatively constant level for both 100% or 50% pot capacity by compensating for the water lost through ET after each gravimetric measurement at 2‐day interval, as has been done by prior researchers (Pellegrino et al., 2004; Ray & Sinclair, 1998). Supplementary irrigation allowance of 50–200 g water/pot was added at different plant developmental stages so as to account for the effects of plant growth on the SWC determination.

### 2.7 Stomatal conductance and leaf chlorophyll content

Leaf stomatal conductance was determined using an AP4 Porometer (Delta T, Cambridge, UK). Measurements were performed on three young but fully expanded leaves once a week (between 10:00 and 12:00), and the mean conductance for each treatment combination was calculated. The leaf chlorophyll content index (CCI) was measured using a CCM‐200 Chlorophyll Content Meter (Optic‐Sciences, Inc.), with measurements being performed on three young but fully expanded leaves.

### 2.8 Leaf stomatal pore and root anatomical features

Microscopic analyses of leaf stomata and root anatomic structures were performed on samples collected on the final date of harvest. Two fully expanded upper leaves were cut from each plant (between 11:00 and 11:30) and immediately immersed in 70% ethanol inside 50 ml centrifuge tubes until they were analyzed. After 2 weeks, the preserved leaf tissues were washed with deionized water three times, added to 40 ml of concentrated sodium hypochlorite, and left to stay for approximately 4 h. The leaf tissues were re‐washed with deionized water, and fresh 70% ethanol was again added onto the samples. This clearing process removed the chlorophyll from the leaves and rendered them a white visual appearance. The stomata of the cleared leaves were then observed using a digital microscope (Leica DVM6) equipped with the tools to measure the length and width of the stomatal pores. The total stomatal aperture area was calculated using the formula for the area of an ellipse, as

(4) Stomatal aperture area=π×a×b,

where *a* and *b* represent the semi‐major axis (or radius) and semi‐minor axis (or width) of the ellipse, respectively.

Roots were washed by spraying tap water to remove soil debris; then root samples 10 cm in length from the growing tip were cut using a razor, washed in deionized water, and fixed in 70% ethanol until the time for microscopic analysis. Next, thin cross sections were made along the root using a sharp razor blade while holding the root protruding from a small polystyrene sheet in an upright posture. The razor blade was perpendicular to the root axis. Cross sections were removed from the blade and put into a Petri dish of water. Two uniform cross sections of roots per plant were analyzed using digital microscope to examine the root anatomical structures. The diameter of xylem vessels and the total root diameter were quantified and compared between SHS mulched and unmulched plants.

### 2.9 Plant growth, fruit yield, and biomass

The plant height was measured every week during the 98 days experimental period. Tomatoes were harvested and weighed at the time of harvest. During the final harvest, the shoots and roots of each plant were weighed, and then put in paper bags and oven‐dried at 105°C for 4 days to determine their dry mass.

### 2.10 HI and TE

After harvest, the HI was calculated for each plant as the ratio of total fruit yield (fruit mass per plant) to total fresh mass (shoot and roots) per plant, as:

(5) $$\mathrm{HI}=\frac{\text{Total fruit yield}\;}{\text{Total fresh biomass}\;}.$$

The TE of each plant was calculated as the ratio of dry shoot biomass in grams to the amount of water transpired in kilograms, as:

(6) $$\mathrm{TE}=\frac{\text{Shoot}\;\mathrm{dry}\;\text{biomass produced}\;\left(g\right)}{\text{Mass of water transpired}\;\left(\mathrm{kg}\right)}.$$

### 2.11 Data analysis

Data analysis and graphical presentations were performed using Origin Pro Software (2019 version). A three‐way analysis of variance (ANOVA) was used to analyze the effects of soil mulching, irrigation regimes, growth stage (i.e., time), and their interactions on ET. For the variables averaged across time (i.e., time‐independent), a two‐way factorial ANOVA was performed to determine the effects of soil mulching, irrigation regimes, and their interactions. In each analysis, mean comparisons were done using the Tukey test at the *p* < .05 level of statistical significance.

## 3 RESULTS

### 3.1 Plant growth

**FIGURE 1:**
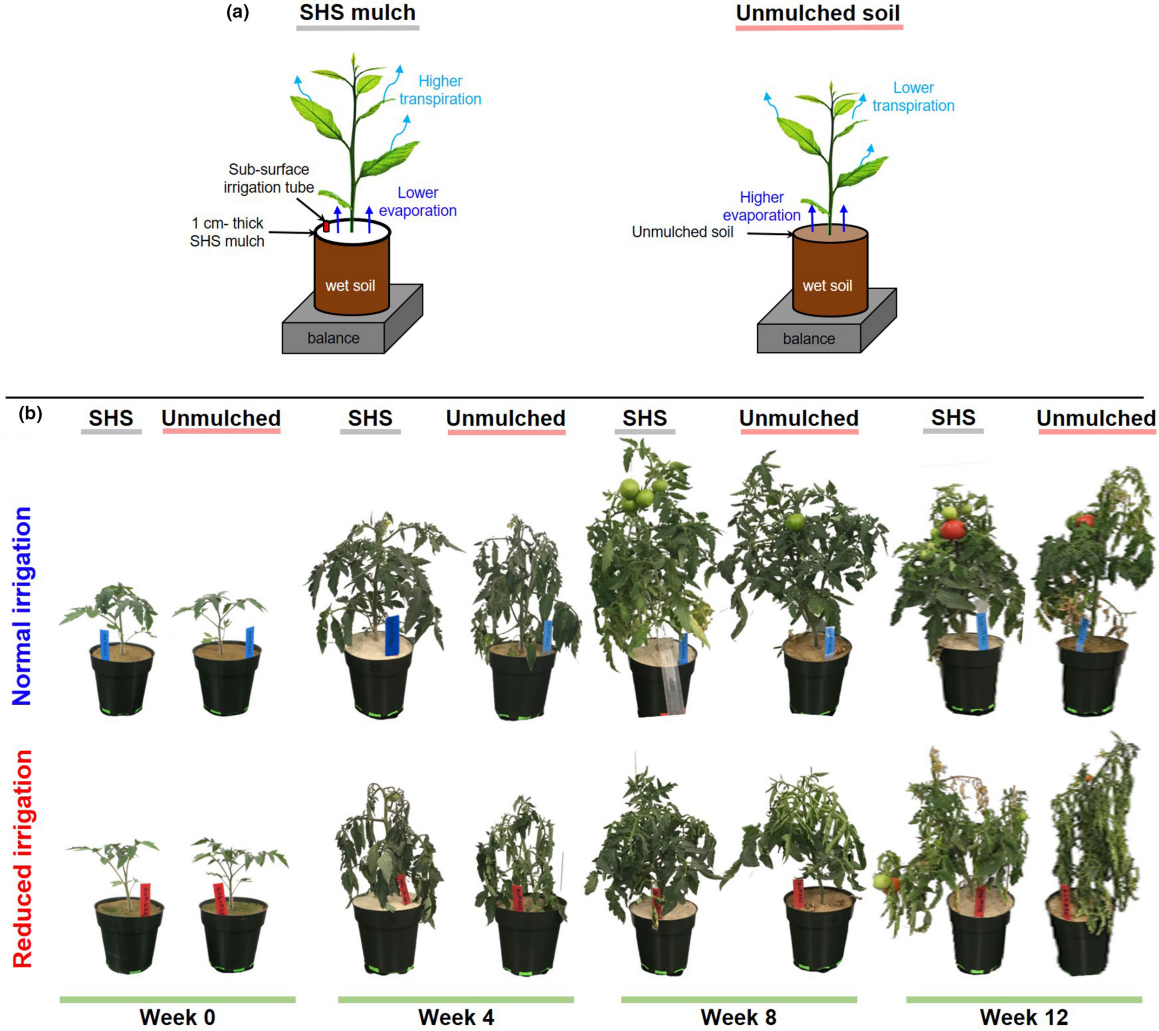
Effects of SHS mulching on plants. (a) Schematics of the evaporation and transpiration pathways and their impacts on plants. (b) Representative photographs of tomato plants grown in controlled growth chambers mulched with 1.0 cm‐thick superhydrophobic sand (SHS) and unmulched soil under normal (**N**) and reduced (**R**) irrigation. Tomato plants were transplanted and grown for 98 days until the final fruit and biomass harvest. Each treatment combination involved four (*n* = 4) replicate plants

Representative snapshots of plants grown under each condition (i.e., mulched and unmulched soils under **N** or **R** irrigation) at different weeks during their growth, from transplanting (week 0) to the final fruit harvest (week 12), are shown in Figure 1. Overall, the plants grown in mulched soils were larger and appeared healthier than those grown in unmulched soil.

### 3.2 Soil water content

**FIGURE 2:**
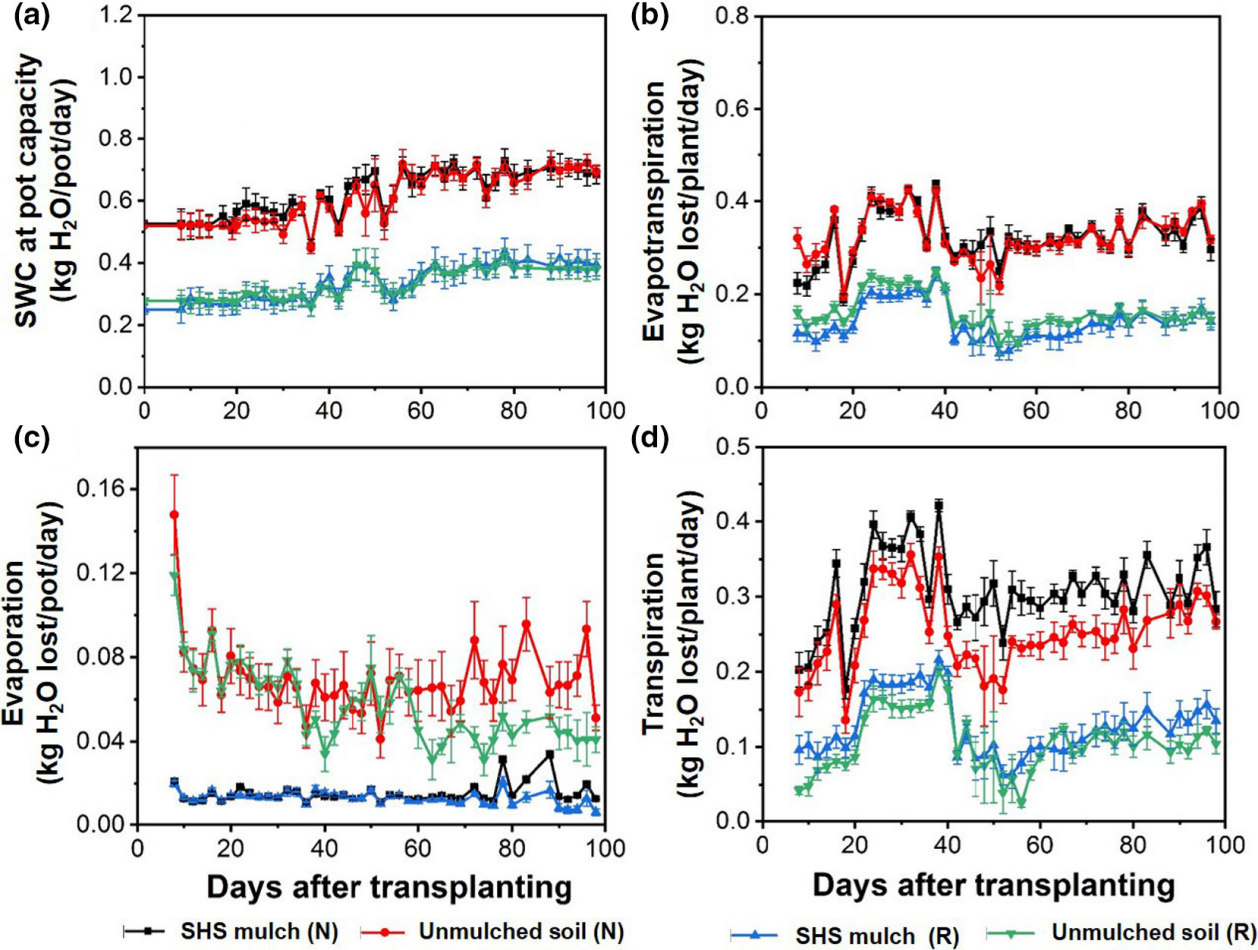
(a) Soil water content (SWC) in pots maintained at pot capacity for each treatment and irrigation regime, (b) daily changes in ET, (c) evaporation from the soil, and (d) transpiration between SHS mulched and unmulched soils under **N** and **R** irrigation strategies throughout plant growth. Each data point is a mean of four (*n* = 4) replicates; error bars are standard errors (±SE) of the means

Daily SWC corresponding to each irrigation level is detailed in Figure 2a. For both **N** and **R** irrigation scenarios (i.e., 100% and 50% pot capacity, respectively), the SWC was slightly increased during days 40–98 to compensate for the increase in plant size and water demand. The fluctuations in SWC below the initial pot capacities observed at five occasions (i.e., between 30 and 50 days) represent 5 days when pot weights were recorded without adding water until the following day due to unavoidable circumstances. However, the plants were not severely stressed on these occasions. When averaged across both mulched and unmulched soils, the mean SWC was 0.62 ± 0.01 and 0.33 ± 0.01 kg/pot under **N** and **R** irrigation, respectively. Thus, we were able to maintain plant growth at 50% of the normal irrigation (i.e., the **R** irrigation strategy).

### 3.3 Evapotranspiration

The daily ET significantly differed between the **N** and **R** irrigation scenarios and accounted for approximately 50% of the daily SWC on average, as shown in Figure 2b. Under **R** irrigation, ET differed between mulched and unmulched soils, notably during days 10–35 and 45–75; no significant differences were found under **N** irrigation.

### 3.4 Soil evaporation and transpiration

Throughout the growth period, the daily evaporation was significantly lower from mulched soil than that in unmulched soil under both **N** and **R** irrigation, as shown in Figure 2c. Consequently, plants in mulched soil had higher daily transpiration than in unmulched soil under both irrigation scenarios, as shown in Figure 2d.

### 3.5 Cumulative ET

**FIGURE 3:**
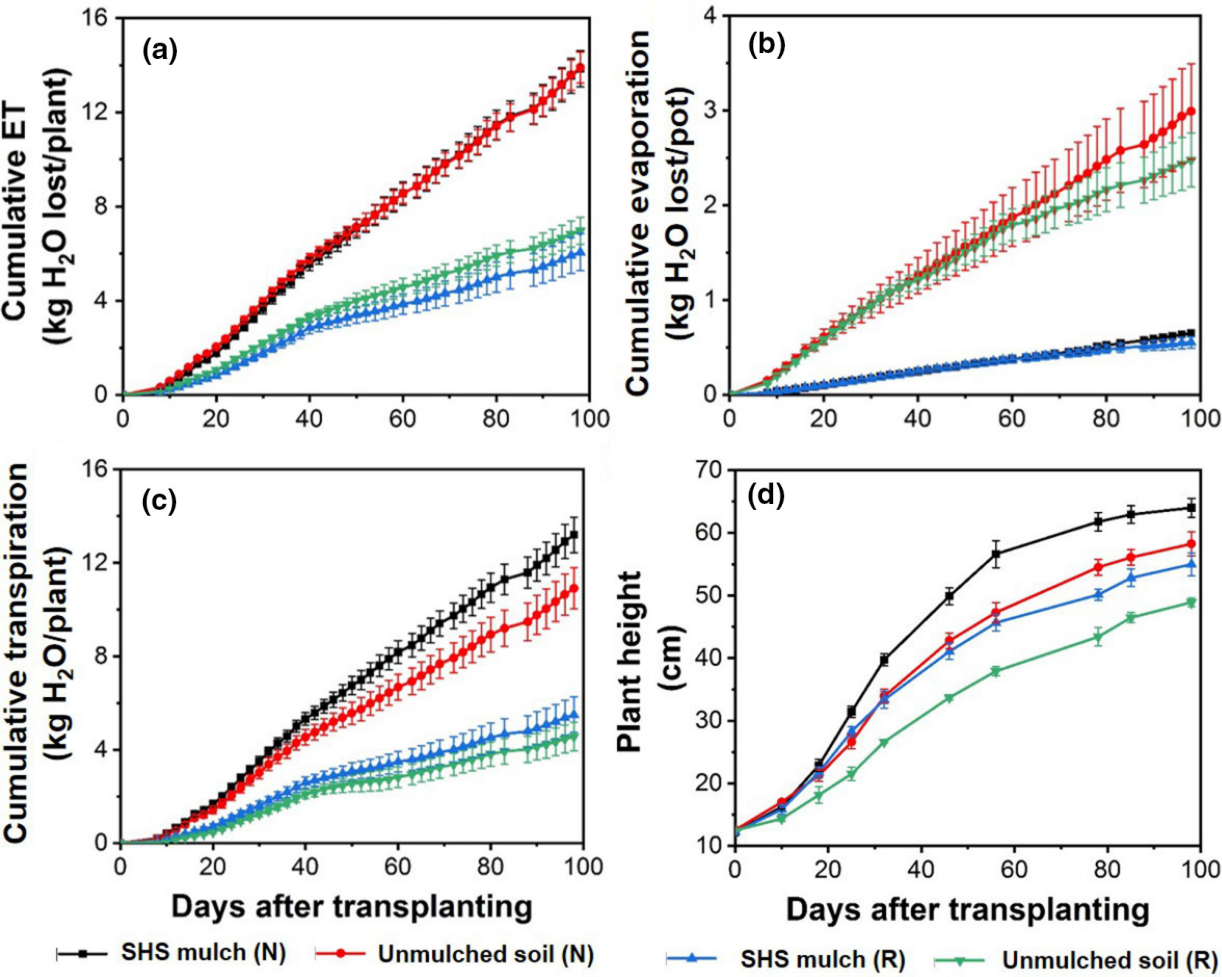
(a) Cumulative evapotranspiration (ET), (b) evaporation, (c) transpiration, and (d) plant height during plant growth under contrasting soil mulching treatments (i.e., SHS mulch vs. unmulched soil) and irrigation regimes (i.e., **N** and **R**). Each data point is a mean of four (*n* = 4) replicates; error bars represent the ±SE of the mean

There was no significant difference in the cumulative ET loss between mulched and unmulched soils under **N** irrigation; both had a mean cumulative ET of 13.9 ± 0.7 kg/plant over the 98 days of observation, as shown in Figure 3a. Under **R** irrigation, the cumulative ET was 13% higher in SHS‐mulched soil than in unmulched soils (with mean values of 6.97 ± 0.57 and 6.06 ± 0.78 kg/plant, respectively).

### 3.6 Cumulative soil evaporation and transpiration

In the mulched soils, the total cumulative evaporation under **N** and **R** irrigation accounted for 5% and 9% of the total ET while transpiration was 95% and 91%, respectively. In unmulched soils, evaporation under **N** and **R** irrigation accounted for 21.5% and 31.5% of the total ET whereas transpiration was 78.5% and 65.5%, respectively (Figure 3b,c). Overall, SHS mulching suppressed evaporation and enhanced transpiration under **N** irrigation by 78% and 17%, respectively relative to the unmulched soils. These effects of SHS mulching were similar in percentage term to those under **R** irrigation but differed quantitatively as the amount of water under **N** was higher than in **R** irrigation.

### 3.7 Plant height

Throughout the growth period, the maximum plant height in SHS‐mulched soil significantly exceeded that in unmulched soil by 9% (*p* = .005) and 11% (*p* < .001) under **N** and **R** irrigation, respectively (Figure 3d). In particular, a more rapid increase in the plant height was observed during 10–45 days following transplanting; after 70 days, plant height started to level off.

### 3.8 Dependence of ET on plant growth stage

Mean ET, evaporation, and transpiration during the days 0–30, 31–60, and 61–98 are compiled in Table 1. Overall, the mean ET, evaporation, and transpiration increased with time; significant differences were present in the mean ET and transpiration between each time‐frame. However, no significant difference was found in the mean evaporation with time (*p* = .392), as evidenced by the three‐way ANOVA results. Significant two‐way interactions were present between mulching and irrigation regimes on transpiration (*p* = .03) and between irrigation regime and growth stage on ET (*p* < .001), evaporation (*p* = .04), and transpiration (*p* < .001).

**TABLE 1:** Effects of soil mulching (M), irrigation regimes (W), growth stage (S)–shown by the three different time periods, and their interactions on mean ET, evaporation, and transpiration

Treatments	ET (kg/plant)			Evaporation (kg/pot)			Transpiration (kg/plant)		
	0–30 days	31–60 days	61–98 days	0–30 days	31–60 days	61–98 days	0–30 days	31–60 days	61–98 days
SHS mulch (**N**)	3.66 ± 0.14	4.89 ± 0.21	5.29 ± 0.22	0.19 ± 0.02	0.21 ± 0.01	0.27 ± 0.01	3.48 ± 0.13	4.68 ± 0.20	5.02 ± 0.22
Unmulched soil (**N**)	3.93 ± 0.14	4.59 ± 0.16	5.34 ± 0.15	0.93 ± 0.11	0.93 ± 0.16	1.12 ± 0.18	3.00 ± 0.21	3.66 ± 0.20	4.23 ± 0.30
SHS mulch (**R**)	1.79 ± 0.14	2.07 ± 0.24	2.20 ± 0.33	0.18 ± 0.40	0.20 ± 0.02	0.18 ± 0.02	1.61 ± 0.13	1.86 ± 0.23	2.02 ± 0.31
Unmulched soil (**R**)	2.24 ± 0.15	2.39 ± 0.17	2.40 ± 0.11	0.92 ± 0.02	0.86 ± 0.08	0.68 ± 0.01	1.32 ± 0.15	1.57 ± 0.22	1.74 ± 0.07

Three‐way ANOVA	ET			Evaporation			Transpiration		
M (df = 1)	3.07 (0.178)			266 (0.00*)			25.0 (0.00*)		
W (df = 1)	702 (0.00*)			5.63 (0.02)			500 (0.00*)		
S (df = 2)	33.0 (0.00*)			1.02 (0.392)			26.0 (0.00*)		
M × W (df = 1)	5.82 (0.202)			0.57 (0.431)			5.28 (0.03)		
M × S (df = 2)	2.05 (0.253)			0.83 (0.139)			1.47 (0.569)		
W × S (df = 2)	15.0 (0.00*)			3.49 (0.04)			7.74 (0.00*)		
M × W × S (df = 2)	0.36 (0.838)			0.29 (0.536)			0.53 (0.489)		

*Note*: Data presented in the upper rows indicate mean values (±SE) of total ET, evaporation, and transpiration at three time periods/growth stages, **S** (0–30, 31–60, and 61–98 days after transplanting) for each treatment combination: SHS mulch under **N** irrigation (SHS mulch‐**N**); unmulched soil under **N** irrigation (Unmulched soil‐**N**); SHS‐mulched mulch under **R** irrigation (SHS mulch‐**R**); and unmulched soil under **R** irrigation (Unmulched soil‐**R**). The lower rows indicate three‐way ANOVA statistics for the effects of M, W, S, and their interactions on total ET, evaporation, and transpiration. Results presented are *F* values followed by their respective *p*‐values in parentheses at *p* < .05 for statistical significance; 0.00* represents *p* < .001.

Abbreviations: df, degrees of freedom; ET, evapotranspiration; SHS, superhydrophobic sand.

### 3.9 Effects of SHS mulching on physiological traits, biomass yield, and fruit yield

**FIGURE 4:**
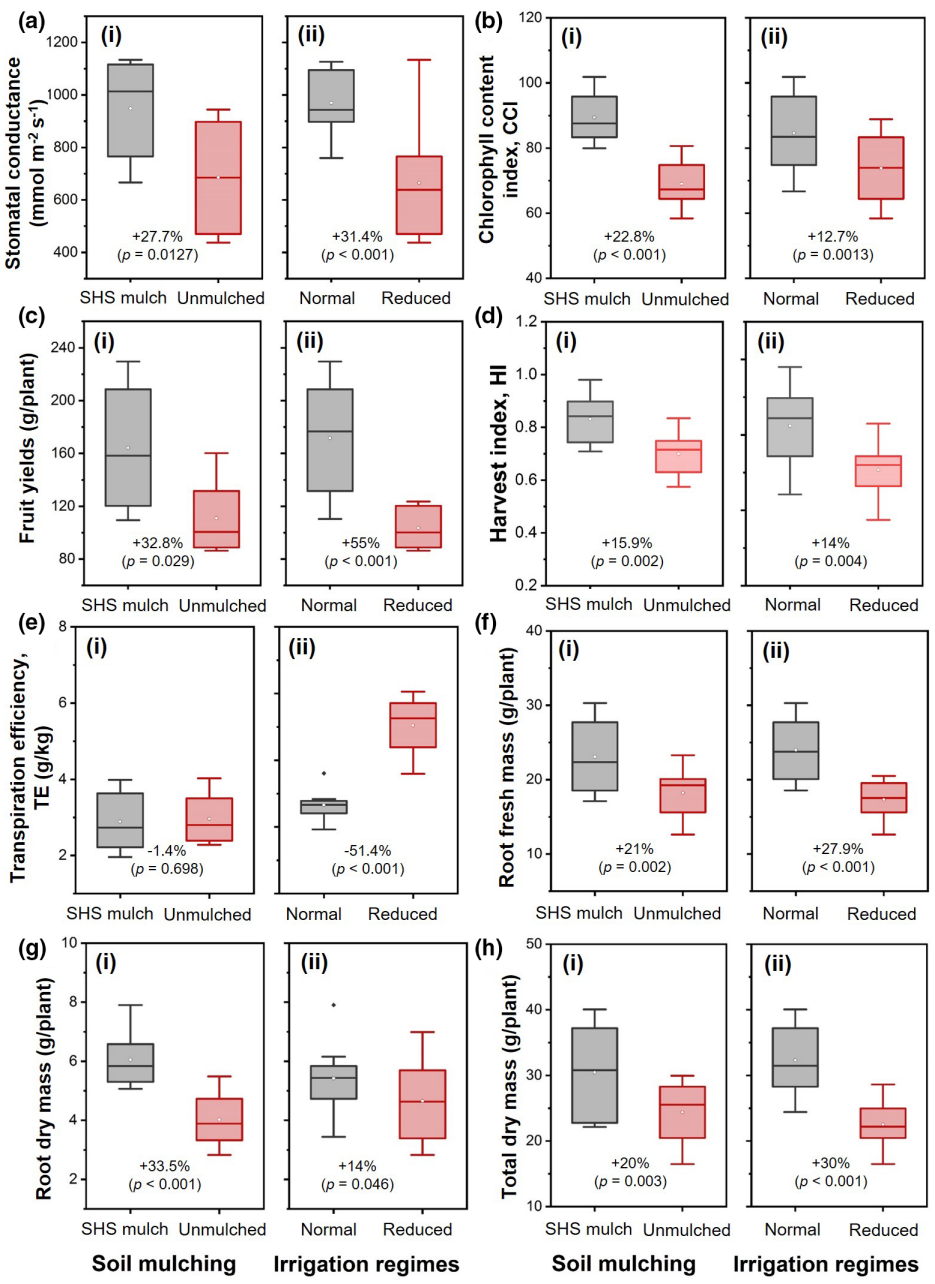
Box plots showing effects of (i) soil mulching (SHS mulch vs. unmulched soil) and (ii) irrigation regimes (**N** vs. **R**) on (a) leaf stomatal conductance; (b) CCI; (c) fruit yields per plant; (d) HI; (e) TE; (f) root fresh mass; (g) root dry mass; and (h) total dry mass per plant. Each box represents the data distribution from eight plants (*n* = 8) with the median along the mid‐line. The white dot inside the box represents the mean value, the upper and lower sections of the box represent the 25% and 75% confidence intervals, respectively, the whiskers on the box represent the 1.5 interquartile range, and dots outside the box indicate outliers. Percentage differences between treatments are presented along with the corresponding *p*‐values derived from two‐way ANOVA at *p* < .05 level of statistical significance

**FIGURE 5:**
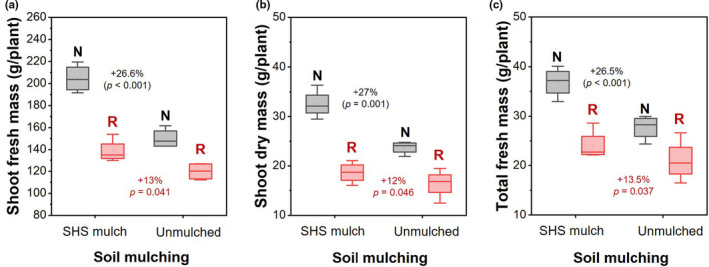
Two‐way interaction effects of soil mulching (SHS mulch vs. unmulched soil) and irrigation regimes (normal, **N** vs. reduced, **R**) on (a) shoot fresh mass, (b) shoot dry mass, and (c) total fresh mass per plant. Each box represents the data distribution from four replicates (*n* = 4) with the median value shown by the mid‐line. The white dot inside the box represents the mean value, the upper and lower sections of the box represent the 25% and 75% confidence intervals, respectively, the whiskers on the box represent the 1.5 interquartile range. Each panel shows percentage differences between SHS mulch and unmulched soils under **N** or **R** irrigation along with the corresponding *p*‐values derived from two‐way ANOVA using *p* < .05 level of statistical significance

**FIGURE 6:**
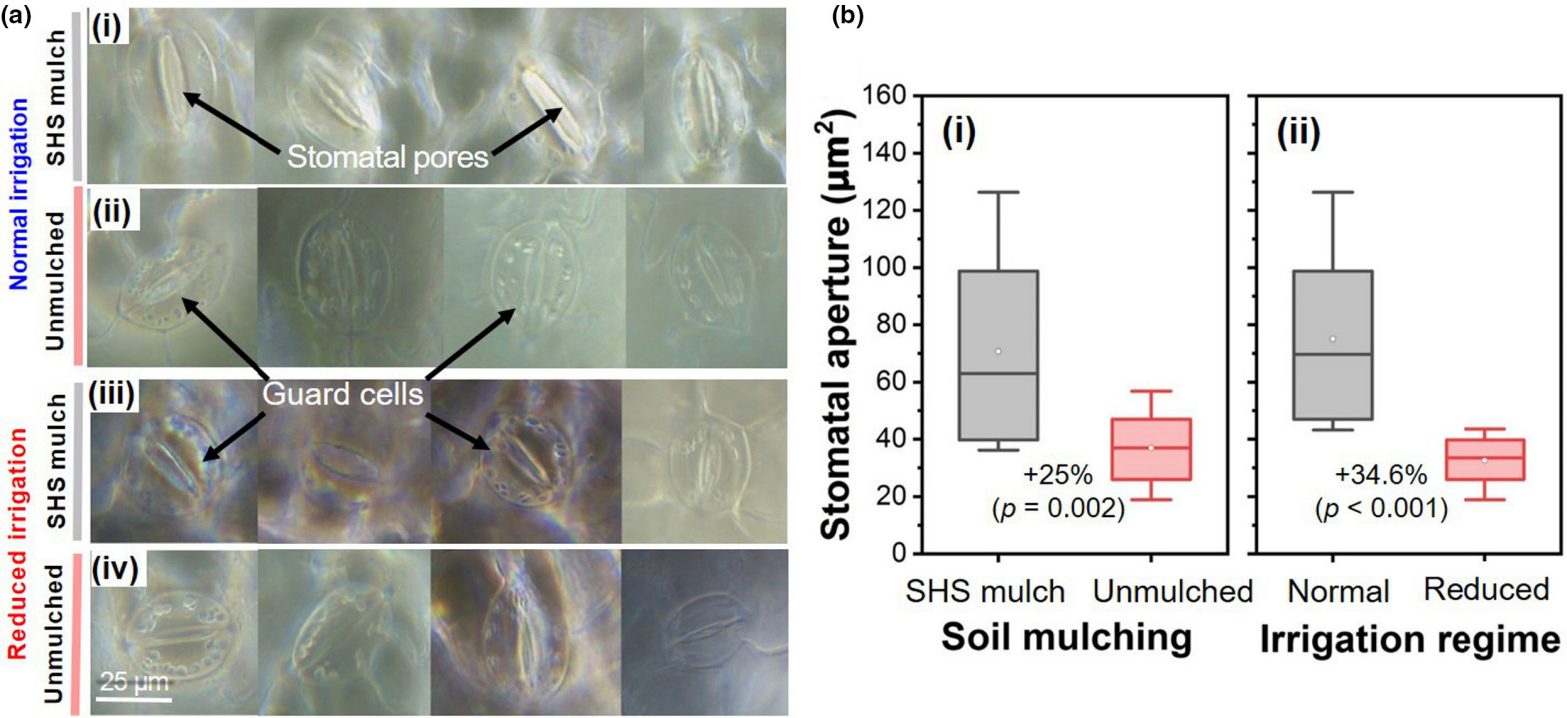
Microscopic appearance of stomatal pores on the final date of plant harvest and main effects of soil mulching and irrigation regimes on stomatal aperture. (a) Leica DVM6 microscopic image of leaf stomata for plants grown under normal, **N** and reduced, **R** irrigation in (i, iii) SHS and (ii, iv) unmulched soils. (b) Boxplots showing the mean stomatal aperture (area) for plants grown in (**i**) SHS and unmulched soil, and (ii) under **N** and **R** irrigation. Each box represents the data distribution from 16 leaf samples (*n* = 16) with the median value along the mid‐line. The white dot inside the box represents the mean value, the upper and lower sections of the box represent the 25% and 75% confidence intervals, respectively, and the whiskers on the box represent the 1.5 interquartile range. Percentage differences between treatments are presented along with the corresponding *p*‐values derived from two‐way ANOVA using *p* < .05 level of statistical significance

**FIGURE 7:**
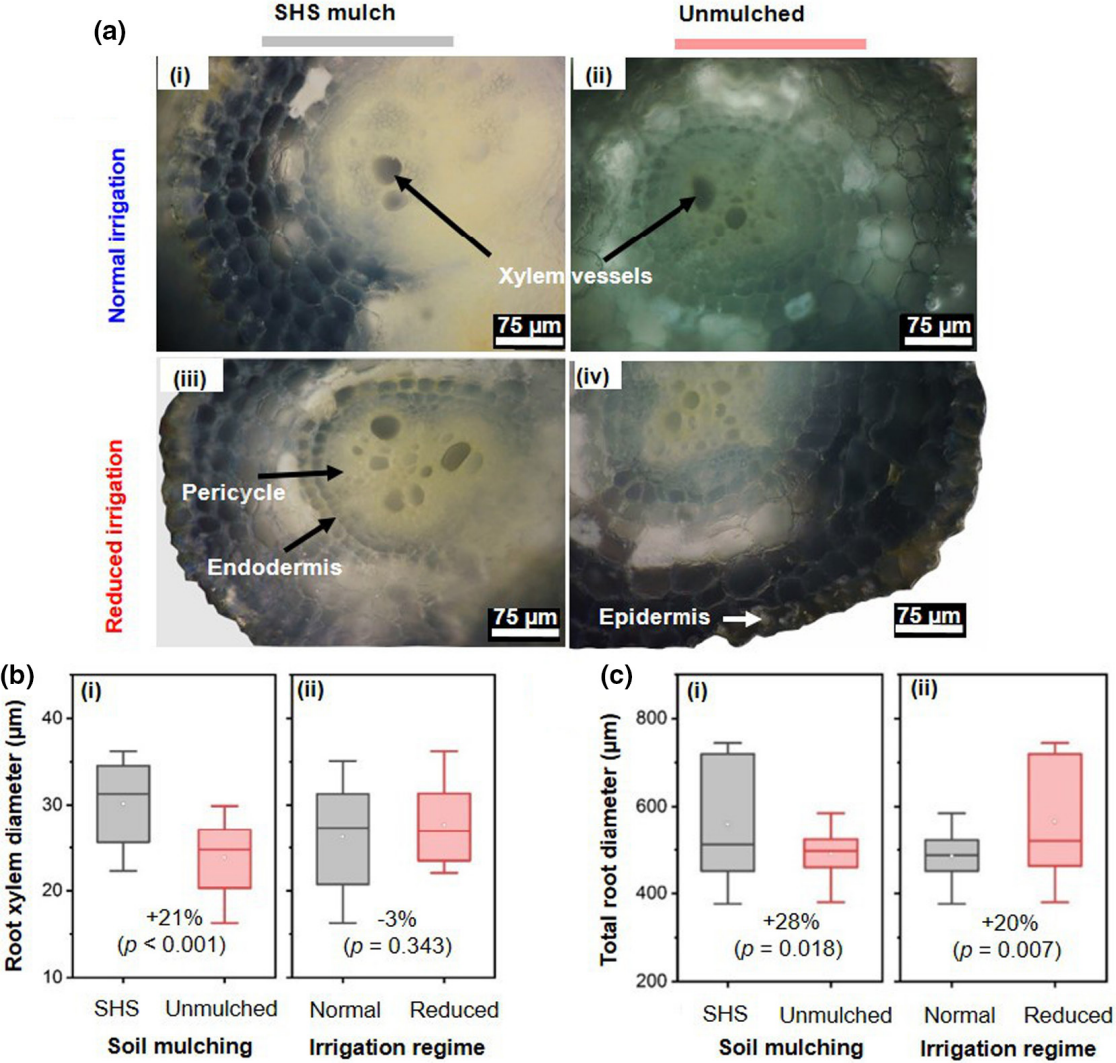
Cross sections of tomato roots from contrasting soil mulching and irrigation scenarios (a, i–iv) and main effects of soil mulching and irrigation regimes (b and c). Box plots showing the effects of (i) soil mulching and (ii) irrigation regimes on (b) root xylem vessel diameter and (c) total root diameter. Each box represents the data distribution from 16 root samples (*n* = 16) with the median along the mid‐line. The white dot inside the box represents the mean value, the upper and lower sections of the box represent the 25% and 75% confidence intervals, respectively, the whiskers on the box represent the 1.5 interquartile range, and dots outside the box indicate outliers. Percentage differences between treatments are presented along with the corresponding *p*‐values derived from two‐way ANOVA using *p* < .05 level of statistical significance

Our two‐way ANOVA demonstrated the effect of SHS mulching was significant on evaporation and transpiration (*p* < .05) but not significant (*p* > .05) on the total ET (Table 2). In addition to the changes in ET, evaporation, transpiration, and plant height demonstrated in Figures 2 and 3, SHS mulching significantly contributed to plant responses such as stomatal conductance and aperture, CCI, fruit yields, HI, fresh mass, dry mass, root xylem vessel, and total root diameter, as presented in Figures 4, 5, 6, 7.

**TABLE 2:** Results of two‐way ANOVA test showing effects of soil mulching (M), irrigation regime (W), and their interactions (M × W) on plant traits measured

Source of variation	df	Total ET	Total evaporation	Total transpiration	Stomatal conductance	Chlorophyll content index, CCI
M	1	1.636 (0.225)	92.7 (0.00*)	12.3 (0.004)	17.5 (0.001)	62.6 (0.00*)
W	1	379 (0.00*)	1.89 (0.193)	232 (0.00*)	23.5 (0.00*)	17.4 (0.001)
M × W	1	1.29 (0.279)	0.89 (0.364)	2.15 (0.169)	1.35 (0.268)	0.013 (0.913)
Error		0.571	0.196	0.849	15,810	26.584

Source of variation	df	Plant height	Shoot fresh mass	Root fresh mass	Total fresh mass	Shoot dry mass
M	1	11.7 (0.005)	51.4 (0.00*)	15.8 (0.002)	53.2 (0.00*)	21.5 (0.00*)
W	1	43.4 (0.00*)	89.9 (0.00*)	29.9 (0.00*)	111 (0.00*)	80.3 (0.00*)
M × W	1	4.41 (0.425)	12.5 (0.004)	3.07 (0.105)	11.2 (0.00*)	7.65 (0.017)
Error		6.468	103.561	5.971	114.8	5.618

Source of variation	df	Root dry mass	Total dry mass	Transpiration efficiency, TE	Fruit yields per plant	Harvest index, HI
M	1	20.6 (0.00*)	14.1 (0.003)	0.16 (0.697)	6.19 (0.029)	15.6 (0.002)
W	1	2.91 (0.114)	36.2 (0.00*)	45.1 (0.00*)	23.3 (0.00*)	12.2 (0.004)
M × W	1	0.723 (0.412)	3.50 (0.086)	1.36 (0.266)	1.99 (0.183)	1.04 (0.327)
Error		0.801	10.518	0.1326	1541	0.004

*Note*: Data presented are *F* values from two‐way ANOVA and *p*‐values for both significant (*p* < .05) and nonsignificant results (*p* > .05) given in parentheses; 0.00* represents *p* < .001.

Abbreviations: df, degrees of freedom; ET, evapotranspiration.

### 3.10 Main effects of soil mulching and irrigation regimes

We observed a 27.7% higher stomatal conductance for plants in mulched soils than those in unmulched soil across irrigation regimes (*p* = .00127; Figure 4a‐i). Stomatal conductance was also 31.4% higher under **N** irrigation than under **R** irrigation (*p* < .001; Figure 4a‐ii). Similarly, leaf CCI was 22.8% higher in mulched plants than their unmulched counterparts (*p* < .001; Figure 4b‐i). Irrespective of the mulch treatment, leaf CCI was 12.7% higher under **N** irrigation than **R** irrigation (*p* = .001; Figure 4b‐ii). Fruit yield, defined by the total fruit mass per plant, was 32.8% higher in mulched soil than unmulched soil (*p* = .03; Figure 4c‐i) and 55% higher under **N** irrigation than **R** irrigation (*p* < .001; Figure 4c‐ii).

The relationship between fruit yield and total fresh mass (i.e., the HI) was characterized by 16% higher HI in mulched soil than in unmulched soil (*p* = .002; Figure 4d‐i). Regardless of the mulch treatment, the HI reduced under **R** irrigation by 14% (*p* = .004; Figure 4d‐ii). No significant difference in the ratio of shoot dry mass to total water transpired per plant (i.e., the TE) was found between plants grown in mulched and unmulched soils (*p* = .697; Figure 4e‐i). However, the TE was 51.4% lower under **N** irrigation than under **R** irrigation (*p* < .001; Figure 4e‐ii).

In terms of biomass allocation, plants in mulched soil had 21% more root fresh mass than those in unmulched soil (*p* = .002; Figure 4f‐i); plants under **N** irrigation had 28% more root fresh mass than their **R** irrigation counterparts (*p* < .001; Figure 4f‐ii). Root dry mass was 33.5% higher in mulched soil than in unmulched soil (*p* < .001; Figure 4g‐i), and was 14% higher under **N** irrigation than under **R** irrigation (*p* = .046; Figure 4g‐ii). Total dry mass in mulched soil increased by 20% over that in unmulched soil (*p* = .003; Figure 4h‐i), while **N** irrigation accounted for 30% higher total dry mass than under **R** irrigation (*p* < .001; Figure 4h‐ii).

### 3.11 Interaction effects of mulching and irrigation regimes

Significant interaction effects of mulching and irrigation were found on shoot fresh mass, shoot dry mass, and total fresh mass per plant. As shown in Figure 5a, shoot fresh mass was 27% higher in mulched soil than in unmulched soil under **N** irrigation (*p* < .001); whereas under **R** irrigation, the shoot fresh mass was higher in mulched soil by 13% than in unmulched soil (*p* = .041). In Figure 5b, shoot dry mass increased in mulched soil by 27% compared with that in unmulched soil under **N** irrigation (*p* = .001); while under **R** irrigation, shoot dry mass was 12% higher in mulched soil than unmulched soil (*p* = .046). Under **N** irrigation, SHS mulching increased the total fresh mass by 26.5% than in unmulched soils (Figure 5c; *p* < .001); whereas under **R** irrigation, the total fresh mass per plant was 13.5% higher in mulched soil than unmulched soil (*p* = .037).

### 3.12 Leaf stomatal status

Microscopy revealed that leaf stomatal pores for plants grown in mulched soils (Figure 6a‐i and iii) were larger than those in unmulched soils (Figure 6a‐ii and iv); the mean stomatal aperture (area) was 25% larger in mulched soils than unmulched soils regardless of the irrigation regime (*p* = .0019; Figure 6b‐i). Furthermore, the mean stomatal aperture was 34.6% higher under **N** irrigation than **R** irrigation (*p* < .001; Figure 6b‐ii).

### 3.13 Root anatomical structure

Finally, root anatomical features were analyzed to determine the shoot–root feedback linkages with the observed trends in ET as a function of mulching and irrigation scenarios (Figures 7a‐i–iv). The xylem vessel diameter was 21% larger for roots grown in mulched soil than unmulched soil (*p* = .001; Figure 7b‐i). However, the difference in xylem diameter under differing irrigation scenarios was not statistically significant (*p* = .343; Figure 7b‐ii). Additionally, total root diameter increased in mulched soil by 28% relative to the unmulched soil (*p* = .0177; Figure 7c‐i), whereas it decreased from **R** irrigation to **N** irrigation by 20% (*p* = .0067; Figure 7c‐ii).

**FIGURE 8:**
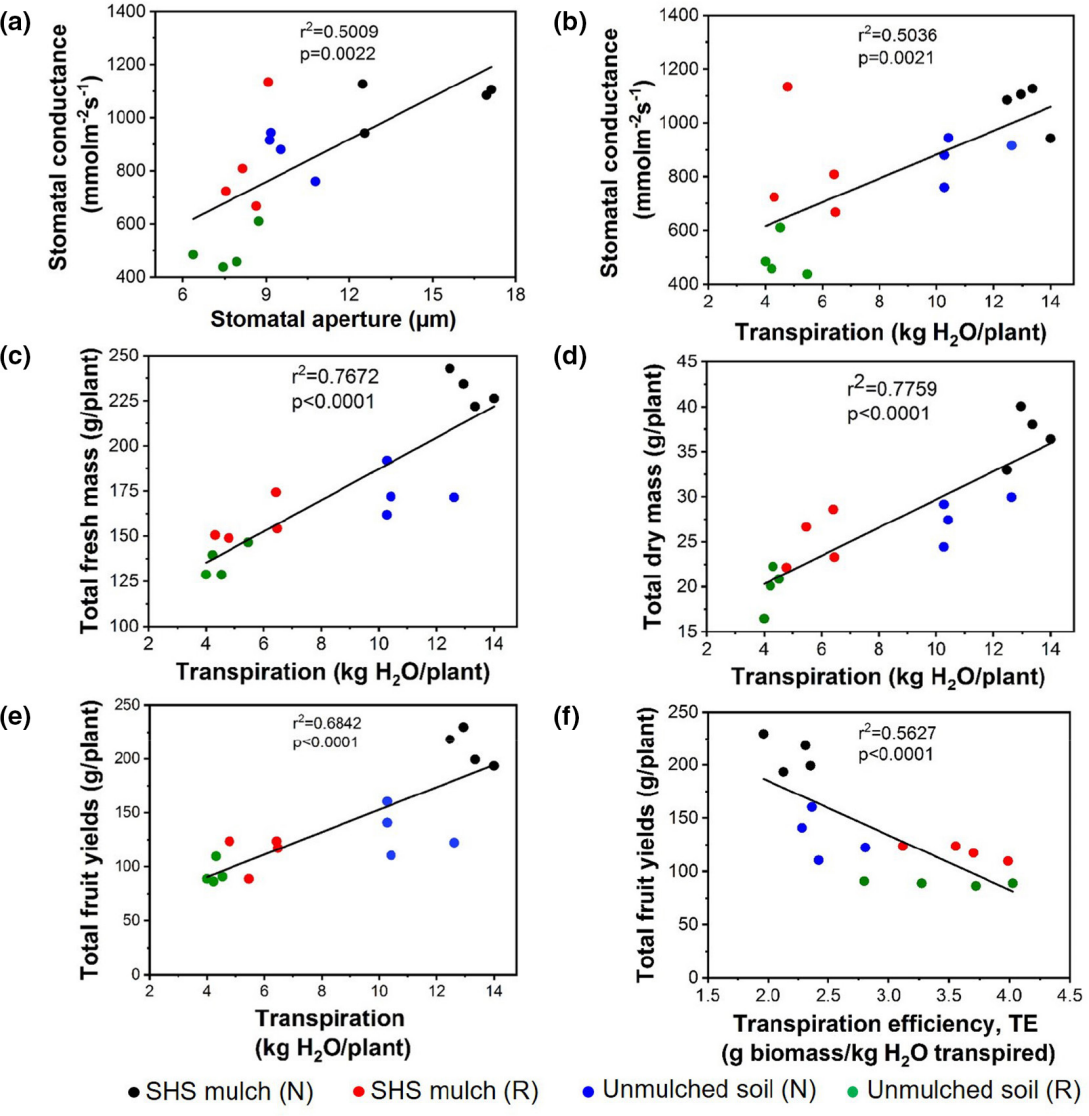
Regression analysis showing the relationship between (a) leaf stomatal conductance and stomatal aperture, (b) stomatal conductance and total transpiration, (c) total fresh mass and total transpiration, (d) total dry mass and total transpiration, (e) total fruit yield per plant and total transpiration, and (f) total fruit yield per plant and TE. Each dot on the graph indicates an individual plant grown in SHS mulch or unmulched soil under **N** or **R** irrigation

To establish the correlation between our results described above, a regression analysis showed significant causal relationships between leaf stomatal conductance and stomatal aperture (Figure 8a), transpiration and stomatal conductance (Figure 8b), transpiration and total fresh mass (Figure 8c), transpiration and total dry mass (Figure 8d), transpiration and total fruit yield (Figure 8e), as well as TE and total fruit yield (Figure 8f). Most parameters were positively correlated except for TE, which had a negative correlation with total fruit yield.

## 4 DISCUSSION

Here, we draw together our experimental results to explain the mechanistic insights into the effects of SHS mulching on phenotypic responses in tomato plants. We start with the effects observed above‐ground followed by those below‐ground and then their interrelationships.

SHS mulching significantly reduced water evaporation from the soil and enhances SWC, which in turn promoted transpiration fluxes (Figures 2 and 3). The enhanced transpiration is attributed to the high stomatal conductance arising from the enlargement of the stomatal aperture in the mulched plants (Figures 4 and 6). This increase in stomatal pore sizes occurred through the active adjustment of the turgor pressure within their guard cells due to higher SWC (Blatt, 2000). Consequently plants' ability for gas‐exchange (e.g., enhanced CO_2_ uptake) and photosynthesis increased; these effects have been noted by other researchers (Bertolino et al., 2019; Condon et al., 2002; Putra et al., 2012).

SHS‐mulched tomato plants had larger xylem vessels and root diameters than the unmulched plants (Figure 7), which is consistent with a previous study on the effects of plastic mulching on maize plants (Zhan et al., 2019). Larger xylem vessels and root diameter boosted the water‐use efficiency, the TE, and the shoot biomass under **N** and **R** irrigation scenarios (Figures 4 and 5). These findings are also in agreement with previous studies on wheat plants (Kadam et al., 2015; Larson & Funk, 2016). Generally, large xylem vessels and root diameter can enhance the hydraulic conductivity of the soil water in the root zone (Bertolino et al., 2019; Comas et al., 2013; Niklas, 1985; Odokonyero et al., 2017), which has a direct influence on shoot responses (Odokonyero et al., 2017; Tardieu & Parent, 2017; Zarebanadkouki et al., 2016).

SHS mulching enhanced tomato plants' CCI, which contributed to higher fruit yield, biomass, and HI (Figure 4). These effects necessitate robust synergism in root–shoot hydraulics to conduct nutrients such as nitrogen via the transpiration stream to the leaves. Nitrogen availability in the soil and its uptake impact the total chlorophyll content in plant leaves (Bassi et al., 2018). Thus, the higher the CCI, the more efficient is the plant’s photosynthetic capacity in mulched soil, leading to the increased growth, biomass, and yields (Condon et al., 2004; Haefele et al., 2009).

Next, we point to some observations that require further investigation to be fully explained. We observed a negative correlation between tomato fruit yield and TE (Figure 8f), although higher TE is known to increase crop yields in water‐limited environments (Christy et al., 2018; Condon et al., 2004; Coupel‐Ledru et al., 2016; Rebetzke et al., 2002; Sinclair, 2012; Vadez & Ratnakumar, 2016). We consider that TE is a physiologically complex trait that depends on multiple physiological parameters, including photosynthesis, stomatal conductance, mesophyll conductance (i.e., the diffusion of CO_2_ from sub‐stomatal cavities to the sites of carboxylation in the chloroplasts) (Flexas et al., 2008; Flexas & Medrano, 2002), and other conditions that determine carbon balance and growth in plants (Natarajan et al., 2021). As a result, the conditions responsible for high TE in one situation may be associated with a low TE in another environment (Sinclair, 2012). Next, against common expectation, we did not observe significant differences in the TE of mulched and unmulched soils despite significant differences in the SWC, stomatal aperture and stomatal conductance. Instead, a lower stomatal conductance was associated with a higher TE under **R** irrigation than under **N** irrigation. This could be related to the drought avoidance mechanism whereby plants under **R** irrigation reduce their stomatal opening to optimize water use under the constraint of soil water deficit (Tardieu, 2005). Consequently, higher TE can be attained under **R** irrigation than under **N** irrigation as we observed in our study and also noted by others (Balwinder‐Singh et al., 2011).

Lastly, we discuss the limitations of this study vis‐à‐vis real‐world implications. Obviously, real arid conditions such as in western Saudi Arabia are significantly harsher than the conditions of the present controlled‐environment study. Furthermore, unlike pots, there is no restriction to root growth and volume in the field (Ray & Sinclair, 1998; Ronchi et al., 2006; Wang et al., 2001); and evaporation from pots is affected by the potted area covered by leaves, unlike in the fields where plant area and density are not limited by space (Lu et al., 2018). Here, we draw the reader’s attention to the main results of our 4‐years‐long field trials of SHS mulch on tomato (*S. lycopersicum*) plants (Gallo et al., 2022). These experiments revealed that SHS led to significant reduction in the evaporation fluxes under **N** irrigation (30%–38%) and enhancement in the fruit yields (27%–72%), which is totally in agreement with the present study. Therefore, insights furnished by this controlled‐environment investigation into the interrelatedness of the various phenotypic responses are at least qualitatively relevant. In the future, we plan to quantify the effects of SHS mulching on root growth via noninvasive methods (Brown et al., 1991; Daly et al., 2015; Downie et al., 2012; Zhu et al., 2011), instantaneous leaf gas‐ exchange, and carbon isotope composition (*δ* ^13^C) (Bednarz et al., 1998; Evans et al., 1986; Monneveux et al., 2006) toward a deeper understanding of above and below‐ground processes in controlled environments and field settings.

## 5 CONCLUSION

We investigated the effects of SHS mulching on ET and plant phenotypic traits inside growth chambers under normal and reduced (50% of normal) irrigation scenarios. We found that irrespective of the irrigation regime, SHS mulching significantly suppressed evaporation and enhanced transpiration by 78% and 17%, respectively, compared with the unmulched soils (absolute numbers vary). Mulched plants had larger stomatal aperture (25%) and stomatal conductance (28%), and larger xylem vessel (21%) and root diameter (28%). These effects synergistically boosted root–shoot hydraulics. As a result, mulched plants had superior phenotypic features such as plant height (9%–11%), fruit yield (33%), total fresh mass (27%), and total dry mass (20%), relative to their unmulched counterparts. Taken together, these findings provide mechanistic insights into the beneficial effects of SHS mulching and underscore this technology’s potential for achieving sustainable food security and greening initiatives in arid regions such as the Middle East and beyond.

## CONFLICT OF INTEREST

Himanshu mishra and Adair Gallo Jr. have filed a US patent (US20200253138A1), While Kennedy Odokonyero and Vinicius Dos Santos do not have any competing financai interests.

## Data Availability

All data supporting the findings reported in this study are available in the paper. H. M. and A. G. have filed a US patent (US20200253138A1) for the SHS used in this work, while K. O. and V. D. S. have no competing financial interest in this work.
